# Foreign Language Learners Show a Kinematic Accent in Their Co-Speech Hand Movements

**DOI:** 10.1162/OPMI.a.321

**Published:** 2026-01-15

**Authors:** Hans Rutger Bosker, Marieke Hoetjes, Doenja Hustin, Wim Pouw, Lieke van Maastricht

**Affiliations:** Max Planck Institute for Psycholinguistics, Nijmegen, The Netherlands; Donders Institute for Brain, Cognition, and Behaviour, Radboud University Nijmegen, Nijmegen, The Netherlands; Center for Language Studies, Radboud University Nijmegen, Nijmegen, The Netherlands; Department of Computational Cognitive Science, Research Center for Cognitive Science & Artificial Intelligence, Tilburg University, Tilburg, The Netherlands

**Keywords:** timing, hand movements, speech, prosody, kinematics

## Abstract

Humans typically move and vocalize in a time-synchronized fashion, aligning prominence-lending hand movements to acoustically emphasized syllables. This requires complex coordination. When speaking a foreign language, learners often place prominence on the wrong syllable in a word, which contributes to a noticeable foreign accent. In this pre-registered kinematic-acoustic study, we test whether a foreign accent is present in the timing of co-speech manual movements. Results demonstrate a ‘kinematic accent’ in Dutch learners of Spanish producing Spanish cognates (e.g., Spanish *profe**SOR*** - Dutch *pro**FES**sor*). Dutch learners time the maximum extension of their co-speech movements closer to the prominent syllable in their native Dutch (i.e., on *-fes*), even when acoustically emphasizing the correct Spanish syllable (*-sor*). Conversely, when incorrectly acoustically emphasizing the Dutch target (*-fes*), the maximum extension of their hand movement is attracted to the Spanish target syllable (*-sor*). This reveals competing timing processes between movement and vocalization systems for foreign language learners, demonstrating that not only your spoken accent but also your co-speech manual kinematics may give away your native language.

## INTRODUCTION

Speaking is a complex physical process. Multiple systems work in concert to produce language sounds, including respiratory cycles, vocal-fold vibrations, as well as tongue, velum, jaw, and labial movements (Chandrasekaran et al., 2009; MacNeilage, 2010). Upping the complexity, when speakers produce manual communicative movements, they do so in close temporal synchrony to speech (Kendon, 2004; Krivokapić et al., 2017; McNeill, 1992; Parrell et al., 2014; Rochet-Capellan et al., 2008; Wagner et al., 2014). This coupling between hand movements and speech is so natural that when one is instructed to produce emphasis in one modality, for instance by moving one’s hand up and down, this will unintentionally lead to emphasis in the other modality, speaking, and vice versa (Krahmer & Swerts, 2007; Parrell et al., 2014; Pouw & Fuchs, 2022; Shattuck-Hufnagel & Ren, 2018; Wagner et al., 2014). However, the precise timing mechanisms of hand movements and speech synchrony are poorly understood. It is especially unclear whether manual and speaking movements are governed by a single system or not (Feyereisen, 2017; McNeill, 1992). Answering this question will help resolve a more general conundrum of how peripheral motor and vocal systems are functionally (de-)coupled in human and non-human animals (Berg et al., 2019; Pouw & Fuchs, 2022). Here, we combine knowledge from research on speech perception, second language acquisition, gesture studies, and kinematics to assess how the gestural and articulatory timing mechanisms are synchronized in the context of lexical stress production by foreign language learners.

Stress in this context refers to the greater prosodic prominence of one element compared to similar elements within a linguistic unit. In speech, for instance, prominence is attested at both the phrasal and the lexical level. Prosody can be used at the phrasal level to highlight specific words within an utterance (Ladd, 2008), while at the lexical level, a syllable within a word can be made more salient relative to others (Cutler & Jesse, 2021) via similar acoustic modulations of the relative loudness/amplitude, pitch, and duration of syllables (van Heuven, 2018). For example, in the Spanish word *profe**SOR***, the final syllable is the most prominent one (indicated by bolded capitals). The two levels of acoustic prominence can also overlap, as is the case in the present study, in which words are uttered in isolation, hence acoustic cues to lexical prominence coincide with those to phrasal prominence and are all realized on the same, most prominent, syllable of the word. Prominence in spoken language is a multimodal phenomenon: its production (by speakers) and its perception (by listeners) involves more than just the acoustic speech signal (Bosker & Peeters, 2021). For instance, hand (and head) movements often show an emphasis in impetus (a beat) that temporally aligns with the acoustic peak in the speech signal. This is a well-studied phenomenon known as *multimodal prosody* (Wagner et al., 2014). Research has shown that producing a hand movement can interact with speech production through biomechanics (e.g., Pouw et al., 2021), which offers a reason as to why it is natural to time bodily movements together with acoustically prominent syllables. Furthermore, there is a popular theoretical framework in gesture studies, known as growth-point theory, that suggests that core linguistic processes that are also part of speech formalization are responsible for gesture generation (McNeill & Duncan, 2000).

Foreign language acquisition provides a unique context to investigate multimodal linguistic timing as learners experience cross-linguistic competition in both speech and gesture (Alferink, 2015; Brown & Gullberg, 2008; Kellerman & Van Hoof, 2003). This cross-linguistic competition is known to occur at the level of prominence assignment when the foreign language differs from the native language in typical or allowed stress patterns, and even more so when words are otherwise quite similar between the two languages (Post da Silveira et al., 2014). Since such cognates largely share their segmental phonology with the native language, they appear to function as ‘false friends’ (Edmunds, 2009; Post da Silveira et al., 2014). For example, the relevant languages in the present study, Dutch and Spanish, are similar in that they are both free (lexical) stress languages: A given multisyllabic word must be characterized by one of the syllables being more prominent (at least acoustically) than the others. In both languages, lexical prominence is also phonologically contrastive, hence its placement can determine the meaning of a word while its segments remain the same. Therefore, when a Dutch learner of Spanish pronounces *profe**SOR*** in Spanish, the articulation plan competes with the native Dutch articulation plan *pro**FES**sor*. This competition from the native language at the level of prosody is substantial (van Maastricht et al., 2016a; van Maastricht, Krahmer, et al., 2019) and problematic, often leading to incorrect prominence placement on the native competitor syllable (*-fes*) rather than the correct foreign language target syllable (*-sor*; Kim, 2020; van Maastricht, Hoetjes, & van Drie, 2019).

For foreign language learners, acquiring appropriate multimodal prosody is important since native listeners are sensitive to, and critical of, deviations in prominence conveyed via prosody (van Maastricht et al., 2016b) and manual movements (Tsunemoto et al., 2022; Yin & Cai, 2024). Hence, not only do foreign language learners need to learn to acoustically highlight the correct syllable (*-sor*, not *-fes*, in Spanish *profe**SOR***), but they also have to coordinate their hand movements with prominence production in a timely fashion. How foreign language learners acquire the correct prominence patterns, as well as appropriate hand movement timing, and in which order, continues to perplex researchers. That is, although the existence of speech-gesture coupling is undebated (Wagner et al., 2014), such that gesture and speech undoubtedly occur together in time and are often semantically linked, other cognitive processes presumably also affect this relationship. For example, native language competitors in the form of cognates (tested here) or language-specific encoding of path vs. manner (Brown & Gullberg, 2008) may in some contexts influence the coupling between gesture and speech. Arguably, many contextual factors (i.e., next to native language transfer), such as semantic complexity, frequency effects, and working memory constraints may play a role here, but no model of speech-gesture integration to date has enabled the inclusion of all of these factors. The aim of the current study is not to put forward such a model; instead, this study attempts to describe one particular factor in such a model, namely the influence of one’s native language on temporal motor-speech alignment in a foreign language.

Perhaps, informed by previous work in gesture studies (Kendon, 2004; McNeill, 1992), hand movements and speech go ‘hand in hand’ (So et al., 2009), also in foreign language learning. That is, whenever the acoustic prominence pattern is (in)correct, the timing of an accompanying manual movement is also (in)correct. This reflects a close temporal and semantic alignment between gesture and speech (Wagner et al., 2014), supported by findings that foreign language learners show native language transfer in their gestures (e.g., Gullberg, 2009a, 2009b). Additionally, dynamic approaches to cognition have likewise emphasized that gesture and speech are coordinated as a single system (Kelso et al., 1983; Parrell et al., 2014; Rusiewicz & Esteve-Gibert, 2018).

Yet, gesture-speech alignment is not always guaranteed. Some studies suggest that changes in gesture may actually pave the way for native language development (Iverson & Goldin-Meadow, 2005). Moreover, prior work on biomechanical coupling suggests that the production of effortful hand movements can directly contribute to the realization of acoustic prominence in speech (Pouw & Fuchs, 2022, for an overview). Therefore, perhaps the correct timing of hand movements precedes the realization of accurate acoustic prominence, serving as a helpful temporal anchor for speech production. However, learners might—conversely—also acquire correct acoustic prominence placement first, followed by native-like gestural timing, simply because producing hand movements in communication tends to be optional.

A final alternative could be that the kinematic and speech systems have separate timing regimes that can vary flexibly: sometimes the voice is accurate but the timing of the manual movement is off, and at other times the manual movements are timed appropriately but the spoken prominence is located incorrectly. Indeed, studies showed that there may be bidirectional influences of both native and foreign language in speech and gesture (Brown & Gullberg, 2008).

Therefore, in this pre-registered study, we investigate how adult Dutch learners of Spanish produce multimodal prosody. We combine techniques and knowledge from the areas of cognitive psychology, human movement, gesture studies, phonetics, and foreign language acquisition. Inspired by work on biomechanical gesture-speech coupling, we ask (1) whether a single hand movement (an elbow extension) boosts the acoustic markers of prominence, (2) if a hand movement helps foreign language learners to produce the acoustic prominence on the correct syllable in the foreign language, and (3) whether hand movements made by foreign language learners still show a temporal attraction that reflects their native language (i.e., a ‘kinematic accent’). In the current experiment, we make use of instructed biphasic co-speech hand movements (elbow extension followed by an immediate elbow flexion). These artificial movements are different from hand gestures in so far that they are not spontaneously—and therefore not naturally—produced. Of course, manual gestures are also hand movements, and at least part of the motor control processes overlap between co-speech hand movements and co-speech gestures proper. For example, a co-speech gesture that is similar in form to a co-speech movement will not generate different biomechanics. In sum, while we avoid the term gesture in our paradigm, we are confident that outcomes on co-speech hand movements bear implications for ‘gestural’ motor control processes, similar to other studies (e.g., Krivokapić et al., 2017; Treffner et al., 2008; Zelic et al., 2015).

## METHODS AND MATERIALS

### Participants

Informed by a simulation-based (Kumle et al., 2021) power analysis (see pre-registration: https://osf.io/7dj54), we recruited 26 native speakers of Dutch (73% female, 27% male, *M* age = 20.53, *SD* age = 2.40, handedness: 15% left-handed, 77% right-handed, 8% ambidextrous) who were first-year students of Spanish at Radboud University Nijmegen. Self-reported Spanish proficiency levels by the participants were A1 (*n* = 54%), A2 (*n* = 31%), B1 (*n* = 8%), and B2 (*n* = 8%) according to the Common European Framework of Reference levels (CEFR; A1-C2). Additionally, we recruited 26 native speakers of Spanish (62% female, 38% male, *M* age = 24.04, *SD* age = 9.39, handedness: 4% left-handed, 96% right-handed), all students or staff members at the University of León, for a Verification Test of our acoustically-informed prominence metric (see below). This research has been approved by the Humanities ethical review board of the Radboud University (protocol. nr. 2020-2874).

### Materials

Participants were instructed to read Spanish words out loud from a computer screen, one by one. We used R (R Core Team, 2012) for presenting the stimuli to the participants and for item randomization (see the script on OSF). The material consisted of 96 Dutch-Spanish words that are nearly identical in form and meaning between the two different languages. Because the number of Dutch-Spanish cognates is limited, our stimuli set was not perfectly balanced for the number of syllables in the word or the extent and direction of the mismatch between the position of the most prominent syllable in the native vs. foreign language; see Appendix 1 of the pre-registration (https://osf.io/7dj54) for the full stimuli list and their characteristics. Of these so-called *cognates*, 48 were *matching* in word-internal prominence with their Dutch counterpart (e.g., Dutch *bal.le.**RI**.na* and Spanish *bai.la.**RI**.na*) and 48 were *mismatching* in word-internal prominence with their Dutch counterpart (e.g., Dutch *pro.**FES**.sor* and Spanish *pro.fe.**SOR***). Half of the matching and half of the mismatching cognates contained an orthographic stress mark, which, in Spanish, is indicative of where lexical prominence should be placed (e.g., Dutch *a.ca.**DE**.mi.cus* and Spanish *a.ca.**DÉ**.mi.co*, and Dutch *car.di.o.**LOOG*** and Spanish *car.**DIÓ**.lo.go* respectively). Each word was produced twice by each participant: once without and once with a single up-and-down hand movement.

### Procedure

Participants were welcomed into the lab, a largely empty room in which a camera was positioned at a 45-degree diagonal angle from the speaker. A computer screen, positioned across from the participant, showed the orthographic stimuli one by one. Participants were given written instructions in their native language (i.e., Dutch for the learners of Spanish and Spanish for the native speakers), an opportunity to ask questions, and they signed an informed consent form. Before starting the task, participants were instructed how to perform the target hand movement. They were shown a video example of such a beat movement, critically without any audio or facial articulatory information by masking the face (thus avoiding priming any particular bimodal synchrony). Then, they were asked to practice the production of the hand movement with a ‘matching’ practice word (***VI**rus*). This way, the experimenter could watch the performance of these practice movements to see whether any of the instructions were misunderstood. The experimenter only intervened if the instructions were misunderstood but under no circumstances was any participant instructed how to align their movements to the co-produced speech. The example video and accompanying instructions in Dutch can be seen following this link. Hence, participants were prompted but not instructed, to move their hand in a down (extension) and up (flexion) motion of the lower arm around the elbow joint, along the sagittal axis (i.e., in front of their body, also see Figure online). Participants were asked to stand still in space (to facilitate video recording) and put their other hand behind their back. This particular movement was selected because movements of higher mass body segments lead to greater downstream consequences on the respiratory-vocal system than smaller gestures (Pouw et al., 2025; Pouw, Paxton, et al., 2020). Movement and no-movement conditions were blocked, switching every six trials. Participants were audio-recorded (48 kHz) and video-recorded (60 Hz).

### Post-Processing and Key Measures

Motion tracking was performed in the post-processing phase using the video-based motion tracking software MediaPipe (Lugaresi et al., 2019). We extracted the human pose information from the video frames at a temporal resolution of 50 Hz and spatial resolution of 1080 by 1920 pixels. From this raw pose-tracking data, we constructed a time series with information about our key kinematic variable of interest: the change in the vertical position of the index finger (i.e., the point of maximum extension).

Acoustic measures were extracted from the audio recording after the syllable boundaries were automatically generated by EasyAlign (Goldman, 2011) and manually checked. Once we had temporal estimates for syllable boundaries within each trial, we determined which of the syllables was acoustically most prominent (link to script). Note that foreign language learners produced our speech materials and therefore were expected to be highly variable as to which acoustic cues conveyed word-internal prominence in any given trial, even within speakers (i.e., applying Dutch-like cue weights prioritizing *f*0, Spanish-like cue weights prioritizing duration, or most likely a mix thereof). Consequently, prominence detection based on perceptual judgments from another set of participants is unlikely to accurately indicate the syllable that the speakers themselves intended to emphasize: native Spanish listeners will apply their native prosody knowledge and standards to the learners’ imperfect speech, while asking native Dutch listeners is incompatible with these Spanish materials. While it is true that an acoustically-informed metric also may not reflect the speakers’ prominence intent perfectly, it is at least preferable to human perceptual coding in that it is 100% reliable in its output. Therefore, following a similar approach previously applied to learners’ speech (Coulange et al., 2024), we determined which syllable was most prominent using an acoustically-informed metric based on a normalized composite score of the different acoustic peaks in duration, fundamental frequency (*f*0), and intensity. Duration, pitch, and intensity are all correlates of lexical and phrasal prominence in Spanish (Ortega-Llebaria & Prieto, 2011). This method has the advantage of being more transparent, better replicable, even adaptable to different hypotheses, and less biased than any perceptual measure.

For each syllable, we computed a weighted prominence score *S*:

$$S_{i}=W_{F}\left(F_{i}^{z}\right)+W_{I}\left(I_{i}^{z}\right)+W_{D}\left(D_{i}^{z}\right)$$

where the prominence score *S*_*i*_ for syllable *i* in an utterance is determined by the weights (*W* = *W*_*F*_, *W*_*I*_, *W*_*D*_) multiplied by the z-normalized $F_{i}^{z}$ (peak *f*0), $I_{i}^{z}$ (peak amplitude), $D_{i}^{z}$ (duration) measures and then summed. Each acoustic measure extracted for each syllable is *z*-normalized for the whole utterance so that they are comparable (i.e., can be added). We set all weights *W*_*D*_, *W*_*F*_, *W*_*I*_ to 0.33 in order to equally weigh their contributions to the prominence score in an unbiased manner (cf. Coulange et al., 2024), though different parameterizations are possible. The syllable with the highest score relative to all other syllables in the utterance was then selected as the acoustically most prominent syllable.

Once we had nominated the acoustically most prominent syllable, we also derived a time point estimate of the (local) maximum of the amplitude envelope for the interval of the nominated prominent syllable. Peaks in the amplitude envelope were used as they seem to be most directly tied to the physical impulse of upper limb movements (as compared to *f*0; e.g., Pouw, Harrison, et al., 2020). Note that this maximum of the amplitude envelope is not necessarily the global maximum (i.e., the syllable with the highest amplitude within the word), as we determined the prominent syllable based on three acoustic markers, only one of which was the amplitude.

### Verification Test

To verify the efficacy of our prominence metric, we collected speech recordings from a sample of native speakers of Spanish (see Participants above). These participants were presented with the exact same procedure as the Dutch learners, after which syllable boundaries were generated with WebMAUS (Kisler et al., 2017) and manually corrected. Their speech data was processed following the same analysis pipeline. We assumed that native speakers would generally produce lexical stress accurately in their native language and, as such, that the phonologically stressed syllable of a given word coincided in the vast majority of cases with the acoustically most prominent syllable in speech productions. This was observed to be the case in 70% of trials. We take this as confirmatory evidence for the use of our metric, performing similar to other metrics run on English (Bentum et al., 2024; Ferrer et al., 2015). Considering the vast variability in the use of prosodic cues to lexical prominence (Severijnen et al., 2024), detecting lexical stress through acoustic cues alone is challenging; even native speakers do not show ceiling performance (Yu & Andruski, 2010). Because acoustic emphasis is strongly related but not identical to phonological word stress, we formulate our conclusions throughout this paper with respect to prominence production, not word stress. Finally, next to our pre-registered analyses relying on our prominence metric, an exploratory analysis that was completely independent from acoustic prominence cues demonstrated qualitatively similar outcomes (see below).

## RESULTS

In our confirmatory analyses, we performed Generalized Linear Mixed Model analyses, including random intercepts. The extended results, including the final models, can be found here. All the effects reported met our adjusted alpha of 0.016. We find that, in ‘matching’ cognates, Dutch learners of Spanish acoustically highlighted the correct syllable in 60% of the trials (e.g., correctly saying *baila**RI**na*). This performance is similar to earlier work in learners of English (Coulange et al., 2024). However, in ‘mismatching’ cognates, they produced acoustic prominence accurately only in 53% of the trials (e.g., correct *profe**SOR***), while in 27% they incorrectly highlighted the native language target (e.g., incorrect *pro**FE**sor*), and in 20% of the trials they incorrectly highlighted a different syllable.

Concerning our research questions, we found that producing hand movements indeed increased the intensity of the prominent syllable (increased magnitude of the peak amplitude envelope). This finding corroborates earlier studies revealing biomechanical coupling between hand movements and speech and lends further credibility to our acoustically-informed unbiased prominence metric. Still, the production of manual movements did not affect the other prominence markers *f*0 or duration, in line with previous work (Pouw, Harrison, et al., 2020). There was no significant difference in foreign language prominence pattern production between the movement and no-movement trials, indicating that the production of hand movements did not support learners in producing the target acoustic prominence patterns (compared to not producing hand movements).

**Figure 1.**
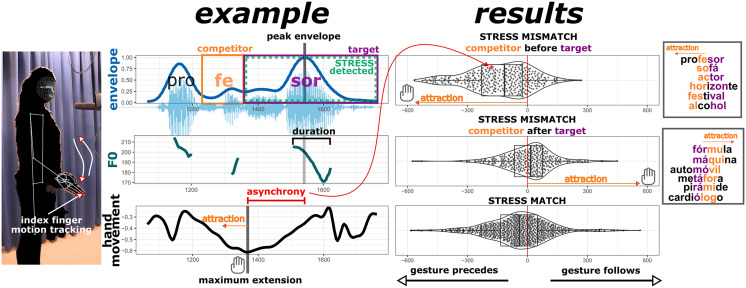
Temporal attraction of hand movements relative to the acoustic intensity peak in acoustically correct word productions. *Note*. **Left panel:** An example is shown for a gesture trial where the Dutch participant pronounced the Spanish word “profesor” (for audio with its spectral profile adjusted to mask identity listen here: https://osf.io/rhegn). In the native language of the Dutch participant, word-internal prominence should be placed on *pro**FES**sor* which competes with the correct target prominence in Spanish *profe**SOR***, making this a ‘mismatching’ item with the competitor syllable (-fes) preceding the target syllable (-sor). The time series reflect the amplitude envelope (given in arbitrary units), *f*0 (Hz), and vertical displacement of the index finger (bodyscaled arbitrary units). The acoustic prominence was detected based on duration, magnitude of the peak envelope, and peak *f*0. The peak envelope serves as the prominence time point, which is typically closely coupled to the hand movement peak (the moment of maximum extension). The asynchrony is the temporal distance in ms between the maximum extension and the peak envelope of the acoustically prominent syllable. **Right panel:** Each subpanel on the right shows the timing of gesture (maximum extension) relative to speech (the peak in intensity of the acoustically prominent syllable) in milliseconds for each trial performed with a gesture. Only trials where prominence was placed on the correct Spanish target syllable (e.g., correct *profe**SOR***) are included here. Positive values indicate that gesture followed speech, and negative values indicate that gesture preceded speech. The upper panel shows gesture-speech timings when the competing syllable of the native language (Dutch) preceded the target syllable of the foreign language (Spanish; e.g., *profe**SOR***, see more examples in the small upper panel on the far right, where orange represents the competitor syllable in the native language and purple the target syllable in the foreign language), the middle panel shows the timings for trials where the competitor syllable followed the target syllable (e.g., ***FÓR**mula*, see more examples in the small lower panel on the far right), and the third, lowest, panel shows the gesture-speech timings of trials where the words in either language carry prominence on the same syllable (e.g., *baila**RI**na*), which serves as a baseline condition. Relative to the trials in this ‘matching’ condition, gesture slightly preceded or followed speech depending on whether (and by how much) the competitor preceded or followed the target syllable. We find this to be the case in the other direction too (not shown here, see Extended Results, section ConfRQ.2C): when speakers incorrectly produce prominence on the competitor syllable, the gesture is slightly attracted to time with the target syllable.

In the correct productions of *matching* cognates that formed our control condition (see the bottom right panel in Figure 1), we observed close manual kinematics-speech synchrony, with the hand’s maximum extension slightly leading the acoustic intensity peak by about −48 ms (in replication of other research in the field of gesture studies, finding gestures to generally slightly precede the stressed syllable; Leonard & Cummins, 2011; Wagner et al., 2014). This finding, well-known from earlier work, lends yet additional support to the accuracy of our prominence metric.

Critically, for correct prominence placement in the *mismatching* cognates, a temporal hand movement attraction occurred in the direction of the competitor syllable of the native language. Specifically, the hand movements *preceded* the acoustic peak slightly but reliably so (model estimate: −88 ms, *SE* = 8.45, *p* < .0001) if the competing syllable of the native language preceded the foreign language target syllable (top right panel of Figure 1). Conversely, hand movements *followed* the acoustic peak when the native language competitor syllable followed the foreign language target syllable (see middle panel of Figure 1; model estimate: 45 ms, *SE* = 8.86, *p* < .0001). Further exploratory analyses showed that these effects could not be attributed to the particular position of the phonologically stressed syllable, since exclusion of items with initial and final lexical stress still led to qualitatively similar results. Also, no evidence was found that individual language proficiency modulated this temporal attraction, presumably due to our sample consisting primarily of beginning learners (see Extended Results).

Moreover, we observed evidence for a *gradient attraction*: the greater the distance between the native language competitor syllable and the foreign language target syllable, the more the gesture was attracted in time (model estimate: −77 ms, *SE* = 14.92, *p* < .0001; see Extended Results). Thus, it is the competing syllable of the native language that temporally attracts the timing of the hand movement, even when the pronunciation itself is accurate (i.e., acoustically highlighting the target syllable correctly in the foreign language). Finally, in response to concerns around the acoustically-defined prominence metric, similar outcomes were observed in an analysis that was completely independent of the acoustic prominence cues. Specifically, evidence for temporal attraction was also observed when calculating the (a)synchrony between participants’ hand gestures and the *phonologically stressed* syllable of the Spanish target word, irrespective of the actually produced prominence cues. That is, the timing of the Dutch learners’ hand movements was significantly earlier in words like *profe**SOR*** (stress-mismatching; preceding native language competitor syllable), and later in words like ***FÓR**mula* (stress-mismatching; following native language competitor syllable), compared to words like *baila**RI**na* (stress-matching; see Extended Results).

Importantly, next to this temporal attraction in the ‘mismatching’ cognates towards the competing syllable of the native language in acoustically *correct* trials, we also found evidence for the opposite: temporal attraction towards the target syllable in the foreign language in acoustically *incorrect* trials. That is, when the Dutch learners of Spanish incorrectly acoustically highlighted the native language competitor syllable instead of the target syllable of the foreign language, their manual movement either preceded (model estimate: −119 ms, *SE* = 10.86, *p* < .0001) or followed (model estimate: 72 ms, *SE* = 15.40, *p* < .0001) the acoustic peak, depending on the location of the foreign language target syllable. Thus, here hand movements are slightly biased to correctly time with the target syllable of the foreign language, even though the pronunciation is incorrect.

Both attraction effects we interpret as a ‘kinematic accent’. For full analysis details, please see the Extended Results in the Supplemental Information, also in light of the pre-registration: https://osf.io/7dj54.

## DISCUSSION

Although producing hand movements did not help our Dutch learners of Spanish to realize acoustic prominence patterns more accurately, we did find that the production of hand movements boosts a stressed syllable’s peak intensity. This extends earlier work on biomechanical influences of upper limb movements on vocalization (Kadavá et al., 2023; Pouw et al., 2021, 2025; Pouw, Harrison, & Dixon, 2020; Pouw, Harrison, et al., 2020; Pouw, Paxton, et al., 2020; Werner et al., 2024) to a novel population and context. The critical finding of this study is that when speakers correctly place their acoustic emphasis according to the foreign language phonology (e.g., correctly saying *profe**SOR*** in Spanish), the hand movement’s emphasis was attracted to time earlier or later depending on where the stress would be in the native language. This temporal attraction was gradient, surfacing more strongly if the distance between the native vs. foreign language stress position was larger. Moreover, the attraction was reliably detected in both the pre-registered analyses (relying on an acoustically-informed prominence metric) as well as in an analysis that was agnostic to the acoustically-produced prominence cues. Together, we take this as evidence for a ‘kinematic accent’ whereby subtle movement-speech temporal asynchrony reveals the speaker’s native phonological system.

Note that this ‘kinematic accent’, measured in terms of temporal asynchrony, is different from a ‘manual accent’ in speakers’ *gestural styles* revealing their linguistic or cultural heritage (e.g., an Italian learner of Dutch producing typically Italian gestures when speaking Dutch; Gullberg, 2009a; Kellerman & Van Hoof, 2003). For example, work on placement event description found that transfer of the native language in gesture was apparent when, despite fluent foreign language speech, the native language was still visible in the semantic information that a gesture provided, or in the lexical alignment of the gesture (Gullberg, 2009b). Our evidence for a ‘kinematic accent’ shows that native language influences can surface at a much lower level in the language production process, affecting the subtle timing of kinematic trajectories of co-speech body movements. However, this should not immediately be taken as evidence that appropriate acquisition of gestural timing lags behind the voice in foreign language learning, because we also found evidence for an opposite pattern. When the acoustic stress was placed on the incorrect native language competitor syllable (e.g., incorrectly saying *pro**FE**sor*), the hands showed temporal attraction toward the correct foreign language target syllable. Thus, we are capturing timing signatures that reflect an unstable, developing, embodied prosody system in which hand movements and speech attract each other (see the found effect of hand movement on intensity) but may also separately be attracted toward either the native or foreign language. In this sense, these findings are in line with previous work showing bidirectional crosslinguistic influences in speech and gesture (Brown & Gullberg, 2008), albeit at a different level in the speech production process.

Taken together, the outcomes show that movement-speech synchrony is determined by multiple factors, including biomechanical constraints, but also the native language system. This has deep implications for understanding the co-development of motor and vocal-articulatory programs. Consider for instance the synchrony between wing beats and vocalizations during flight in vocal-learning compared to non-vocal-learning avian species. Many avian species synchronously couple their wing beats with vocalization units (Berg et al., 2013, 2019). With converging evidence that there are homologous biomechanical constraints of pectoral limbs constraining respiratory-vocal actions (Pouw & Fuchs, 2022), human hand movements similarly affect speech features through biomechanical coupling, in line with current findings. Importantly, it has also been shown that birds who are vocal learners have a more flexible relation between wing beat and vocal coupling than birds who are not vocal learners. Thus, vocal learning animals seem to slightly escape the typical biomechanical constraints, arguably to be able to produce their individual song in a stable manner. We suggest that human language learners too are slightly escaping this natural gesture-speech synchronicity. That is, human movement-speech (a)synchrony cannot be explained in terms of direct biomechanical coupling alone (for a discussion see Pouw & Fuchs, 2022). The timing is not (magically) governed by the biomechanics, but by the speaker through enculturated neural-cognitive control mechanisms that have to take into account the biomechanics in some way (through functional alignment, -counteraction, or by functionally avoiding such biomechanical influences). Indeed, there are different kinds of co-speech gestures that seem wholly outside of the purview of biomechanics and yet show temporal alignment to speech. For example, eyebrow movements do not recruit high-impact physical impulses and can be synchronized with speech (Ambrazaitis & House, 2017, 2022). This raises interesting questions around whether the crosslinguistic influence revealed here would surface all the more strongly in co-speech movements with weaker or no biomechanical coupling to speech.

Our findings suggest that when humans learn to speak a foreign language, novel foreign timing regimes come to compete for prominence-lending actions: both for stress in speech and kinematic emphasis in gesture. In other words, timing competition due to foreign language learning appears to happen on separate speech and manual motor levels. This explains why, in our study, sometimes the voice was on target but the timing of the hand movement was off, while at other times the hands were timed correctly but the voice was off target. Relating these findings to speech-gesture coupling theories (e.g., McNeill, 1992; Wagner et al., 2014), it appears that the temporal aspect of it seems relatively flexible, at least at the low-level language production process that we focus on here. Our results do not dispute that speech and gesture are governed by a single system (McNeill & Duncan, 2000) but this does not preclude the need for coordination given their vastly different time scales. Insofar our findings on hand movements can be applied to the field of gesture studies, it appears that the peaks in gesture and speech may sometimes be attracted to competing targets, at least in contexts of foreign language production.

These findings raise several novel questions for future research. Would there be more pronounced kinematic accents in more naturalistic spoken language, where words are embedded in full sentences with additional language-specific prosodic constraints? Do kinematic accents exist in sign language, such that cognate signs with slight differences between native and foreign sign languages show subtle kinematic biases? Another exciting area concerns whether subtle movement-speech asynchronies in foreign language learners are perceivable to the human eye (Ye et al., 2025). If so, a kinematic accent may directly impact native listeners’ stress perception (Rohrer et al., 2025) and/or the perception of a speaker’s foreign accent. Machine synthesis of gestures in robotics and virtual avatars (Yoon et al., 2022), and possible machine classification of language proficiency might further become more sensitive to kinematic patterns of gestures that may be attuned to speaking a certain language.

## CONCLUSION

To speak a foreign language like a native speaker is extremely difficult for most adult learners. This study contributes to the answer to why this is the case. To ‘speak’ a language is a physical affair that lives through our subtlest of movements. Mastering a language requires the gradual alignment of multiple bodily systems in accordance with new linguistic norms (Thibault, 2020). Novice learners will only achieve this from time to time as the language starts to inhabit their doings.

## ACKNOWLEDGMENTS

We would like to thank Max Ploemen for his help in collecting the data and the anonymous reviewers for their constructive feedback.

## FUNDING INFORMATION

Lieke van Maastricht and Marieke Hoetjes received financial support from the Centre for Language Studies (RG2020-22, RG2021-6, RG2021-34), which is kindly acknowledged. Wim Pouw was funded by a VENI grant (grant nr. VI.Veni 0.201G.047; PI Wim Pouw). Hans Rutger Bosker was funded by an ERC Starting Grant (HearingHands, 101040276; PI Hans Rutger Bosker) from the European Union. Views and opinions expressed are however those of the author(s) only and do not necessarily reflect those of the European Union or the European Research Council. Neither the European Union nor the granting authority can be held responsible for them.

## AUTHOR CONTRIBUTIONS

H.R.B.: Conceptualization; Formal analysis; Investigation; Methodology; Writing – original draft; Writing – review & editing. M.H.: Conceptualization; Formal analysis; Investigation; Methodology; Writing – original draft; Writing – review & editing. D.H.: Data curation; Formal analysis; Investigation; Writing – original draft; Writing – review & editing. W.P.: Conceptualization; Data curation; Formal analysis; Investigation; Methodology; Writing – original draft; Writing – review & editing. L.v.M.: Conceptualization; Data curation; Formal analysis; Investigation; Methodology; Writing – original draft; Writing – review & editing.

## OPEN DATA STATEMENT

The R-markdown code of the Extended Results and pseudonymized data for reproducing the statistical analysis and the primary data are from GitHub. The processing scripts are available on the Github repository that also archives the pre-registration materials (https://github.com/WimPouw/StressInMotion/). The identifiable audio and video data will be available upon request via a Radboud Data Sharing Collection.
